# Community-Directed Bacterial Sexually Transmitted Infection Testing Interventions Among Men Who Have Sex With Men: Protocol for an E-Delphi Study in Toronto, Canada

**DOI:** 10.2196/13801

**Published:** 2019-07-04

**Authors:** Ann N Burchell, Ryan Lisk, Anna Yeung, Jayoti Rana, Jean Bacon, Jason Brunetta, Mark Gilbert, Dionne Gesink, Ramandip Grewal, Charlie B Guiang, Michael Kwag, Carmen H Logie, Leo Mitterni, Rita Shahin, Darrell HS Tan

**Affiliations:** 1 Centre for Urban Health Solutions Li Ka Shing Knowledge Institute St Michael's Hospital Toronto, ON Canada; 2 Department of Family and Community Medicine St Michael's Hospital Toronto, ON Canada; 3 ACT Toronto, ON Canada; 4 Ontario HIV Treatment Network Toronto, ON Canada; 5 Maple Leaf Medical Clinic Toronto, ON Canada; 6 BC Centre for Disease Control Vancouver, BC Canada; 7 Dalla Lana School of Public Health University of Toronto Toronto, ON Canada; 8 Hassle Free Clinic Toronto, ON Canada; 9 Community-Based Research Centre Vancouver, BC Canada; 10 Factor-Inwentash Faculty of Social Work University of Toronto Toronto, ON Canada; 11 Toronto Public Health Toronto, ON Canada

**Keywords:** sexual and gender minorities, sexually transmitted diseases, community-based research, mass screening, patient acceptance of health care

## Abstract

**Background:**

HIV-positive and HIV-negative (gay, bisexual, and other) men who have sex with men (MSM) have experienced a dramatic increase in bacterial sexually transmitted infections (STIs)—syphilis, gonorrhea, and chlamydia. STI testing and treatment mitigate adverse health outcomes and substantially reduce transmission; yet, testing rates remain below recommended levels. Innovation is needed to produce the required increases in testing levels, frequency, and the use of appropriate testing technologies in ways that are engaging, nonstigmatizing, and acceptable to men.

**Objective:**

The aim of this study is to build consensus with regard to interventions with the greatest potential for improving local STI testing services for MSM communities in Toronto, Canada.

**Methods:**

Following a literature review of evidence regarding the effectiveness of novel testing interventions, and focus groups, and surveys to describe local barriers and facilitators of testing among MSM, we will conduct a Web-based, modified Delphi study (e-Delphi). We will form expert panels of community members and STI test providers. Panelists will rate potential interventions in terms of their priority, using a 7-point Likert scale from *definitely not a priority* to *definitely a priority*. They will also rank their preferences by selecting their top 3 preferred interventions. Surveys will be distributed in 3 rounds, with feedback on the distribution of responses from preceding rounds provided in rounds 2 and 3. We will define consensus as having ≥60% (18/30) members indicate a preference within 2 adjacent response points. Qualitative data on disagreements will be obtained using open-ended text responses to explain for ratings and rankings that are different from the majority.

**Results:**

On the basis of a literature review and identification of barriers and facilitators to STI testing among community members and test providers in Toronto, we have selected 8 potential interventions for inclusion in the e-Delphi panel surveys. These include 4 interventions that streamline STI testing for asymptomatic individuals, 2 interventions that are targeted at clients and 2 interventions that are targeted at providers.

**Conclusions:**

Findings will provide community direction for informed decision making regarding the implementation of STI testing interventions in this setting. They will characterize the intervention climate for innovation to STI testing services, including perceived needs for changes to test delivery, relative priorities for change, and readiness for implementation. These methods may be transferable to other urban jurisdictions experiencing similar epidemics and for other contexts where stakeholder input is needed to manage sensitive areas of concern.

**International Registered Report Identifier (IRRID):**

PRR1-10.2196/13801

## Introduction

**Delphi Studies**

Delphi studies are a valuable approach for building consensus around an issue where little knowledge or agreement previously existed [1]. They use “structured anonymous communication between experts…to gather consensus perspectives about an issue or topic that can then be…used to inform decision making” [2]. Traditionally applied using an *in person* format, this design is increasingly being adapted for Web-based environments. Briefly, the Web-based modified Delphi study (*e-Delphi*) involves rounds of Web-based questionnaires in which experts are asked to provide their opinion on particular topics [1,3]. Initially this is done independently, but in subsequent rounds, experts are made aware of the opinions of the group when making their decisions, with the goal of reaching consensus. The key features of the e-Delphi methods are that they are iterative and anonymous, which are particularly beneficial for community-based and patient-oriented research [2,4]. Anonymity and the Web-based format encourage opinion sharing from all panel members, thus preventing dominant individuals from controlling discussion; this is important within hierarchical environments involving the health care system [2,4].

**Bacterial Sexually Transmitted Infections**

We will adapt the e-Delphi method to learn community perspectives to address a pressing health care system issue in our setting: the rise of bacterial sexually transmitted infections (STIs)—specifically syphilis, gonorrhea, and chlamydia. These infections pose a heavy burden on population health, with most cases occurring among HIV-positive and HIV-negative gay, bisexual, and other men who have sex with men (MSM) [5-9]. Untreated syphilis may progress to neurosyphilis, in which symptoms such as meningitis or dementia may develop [10]. Globally, public health agencies are pressing for increased vigilance of antibiotic-resistant gonorrhea strains [11,12]. Certain serovars of Chlamydia trachomatis may cause Lymphogranuloma venereum with painful proctitis and rectal bleeding. Unlike HIV, these STIs can easily transmit via oral sex [13]. In 2014, there were 109,263 chlamydia, 16,285 gonorrhea, and 2357 syphilis cases reported in Canada, much greater than a decade earlier [14]. The true counts are *even higher*, as many cases are asymptomatic and go unreported. Gonorrhea and syphilis rates have dramatically increased among males in the province of Ontario, with nearly all syphilis cases and approximately 40% of gonorrhea cases among MSM and >40% of syphilis cases among HIV-positive MSM [15,16]. We have documented that 23% of HIV-positive MSM have had syphilis, and new infections occur at minimum rates of 1 gonorrhea, 1 chlamydia, and 4 syphilis cases per 100 person years [5-8]. Most cases occur in the city of Toronto, with no signs of a decline [17-19]. Within Toronto, the syphilis epidemic is mature and not restricted to a core sociodemographic group among MSM [8,20], requiring broadscale approaches for control.

STI testing and treatment could mitigate adverse health outcomes and substantially reduce population-level transmission among MSM [21]. However, innovation is needed to produce the required increases in testing levels, frequency, and the use of appropriate testing technologies in ways that are engaging, nonstigmatizing, and acceptable to men. Canadian STI Guidelines recommend annual screening for bacterial STIs among sexually active MSM and as frequently as every 3 months for individuals at *ongoing risk for STIs* [13]. Unfortunately, there are suboptimal levels of STI testing and frequency of testing among MSM in Toronto. STI testing patterns are best known for HIV-positive MSM. In 2009, 55% had tested for syphilis, on average, once per year [8]. As of 2013, we observed only a modest increase to 64% being tested annually, with a few testing more frequently than once per year [22]. Testing rates for chlamydia and gonorrhea are lower than those for syphilis [7,23]; from 2010 to 2013, only 25% of HIV-positive MSM tested annually for genital infection using urine-based tests. Few MSM undergo extragenital testing for gonorrhea and chlamydia, despite Canadian and international guidelines [7,12,23-27]. Without rectal and pharyngeal tests, 71% to 100% of cases will be missed [28,29].

We describe herein our plans to conduct an e-Delphi study as part of a larger mixed-methods study that aims to identify bacterial STI testing interventions for implementation and evaluation among MSM in Toronto. We will assemble 2 expert panels: the first with community members with lived experience as MSM seeking STI testing, and the second with health care providers and public health professionals with expertise in providing STI testing for MSM communities in our setting. Our objective is to build consensus regarding intervention(s) with the greatest potential for improving local STI testing services.

## Methods

All procedures have been reviewed and approved by the research ethics boards of St Michael’s Hospital, Toronto, and the University of Toronto.

### Setting

Toronto is a metropolitan city with a population size of 2.71 million in the province of Ontario [30]. All residents with citizen, permanent resident, refugee, and refugee claimant status have access to provincial or federal health insurance for medically necessary services. STI testing services are available from a variety of sources, including primary care practices, specialist services, or dedicated sexual health clinics.

### Knowledge Synthesis to Select Candidate Interventions

To select STI testing interventions for primary inclusion in the e-Delphi panel, we undertook a review of the published literature [31]. To further refine the interventions, we conducted focus groups with MSM STI testing clients [32] and surveyed health care providers [33] in Toronto. Briefly, the focus groups were conducted with HIV-positive, HIV-negative, and trans-identified men (of any HIV serostatus) to identify barriers and facilitators to bacterial STI testing. Health care providers were surveyed about their current practices, barriers, and attitudes to improve bacterial STI testing rates. Manuscripts for these findings are in preparation.

#### Literature Review

For our literature review, systematic reviews published in 2016 were used as a baseline and updated. These reviews summarized evidence for the effectiveness of STI control interventions, including screening in and outside clinic-based settings published in 2000 or after [24,34]. In addition to repeating the 2016 searches, we expanded literature searches in MEDLINE up to April 2017 using the following keywords: sexually transmitted diseases/STI, chlamydia, gonorrhea, or syphilis. Inclusion criteria for our search were that the article described an intervention aimed at increasing bacterial STI testing; used high-income country settings in urban or semiurban cities; had a study population that included men; and used a study design that was either a trial with a comparison group (controlled, uncontrolled, or pre-post historical controls) or an observational design if it was set in Canada, focused on MSM, or described a Web-based STI testing service. Publications were ineligible if they included only women or heterosexual couples or if they were a study protocol.

Next, we classified the interventions into 3 categories: (1) streamlined testing for asymptomatic individuals, (2) interventions targeted toward clients, and (3) interventions targeted toward providers (Table 1). We use the term *clients* to refer to users of STI testing services, whether or not they are experiencing signs or symptoms of an STI. Using the same strategy as Taylor et al [34], examining outcomes in increasing the proportion tested or increasing frequency of testing, interventions with a comparison group were categorized as very effective (absolute difference (AD) ≥20% or relative difference (RD) ≥100%), moderately effective (AD 5%-19% or RD 10%-99%), or ineffective (AD <5% or RD <10%). Classifications and categorizations were done by JR and verified by ANB. A complete list of the publications used for the final selection of interventions can be found in Multimedia Appendix 1.

The investigators then reviewed the above findings in a series of meetings and selected promising interventions for the Toronto setting to be included in the Delphi panel exercise. Our selection focused on novel approaches for testing rather than efforts that would reinforce existing STI test practices (eg, patient or provider education alone). To minimize respondent burden for panelists, choices are limited to 6 (for community panelists) or 8 (for provider panelists) intervention options.

**Table 1:** Categories of interventions.

Category	Definition
Streamlined STI^a^ testing for asymptomatic individuals	Interventions that focus on testing asymptomatic individuals with a focus on collection of specimens and reducing the time patients spend in clinics
Client-targeted STI testing interventions	Interventions that are targeted at clients to increase client engagement in STI testing
Provider-targeted STI testing interventions	Interventions that are targeted at health care providers to increase provision of STI testing

^a^STI: sexually transmitted infection.

### Recruitment of E-Delphi Panelists

In our application of the e-Delphi method, the term *expert* is meant to include persons with lived experience alongside health care professionals. We will form 2 panels: the first with community members with lived experience as MSM, seeking STI testing in Toronto (*Community Experts*), and the second with health care providers and public health professionals with expertise in providing STI testing for MSM communities in Toronto (*Provider Experts*). We opted to recruit these 2 panels separately, rather than combined, as it was of interest to identify differences in prioritized interventions between the 2 groups, if these exist, rather than forcing consensus between community and provider experts.

To be eligible for the Community Panel, candidates (1) must be a cis- or trans-identified man aged 18 years and older, living in Toronto, and who has sex with men in the preceding 18 months and (2) must have sought and/or underwent STI testing in Toronto in the preceding 18 months.

To be eligible for the Provider Panel, candidates must have a minimum of 1-year experience providing STI testing and management care in Toronto.

**Figure 1:**
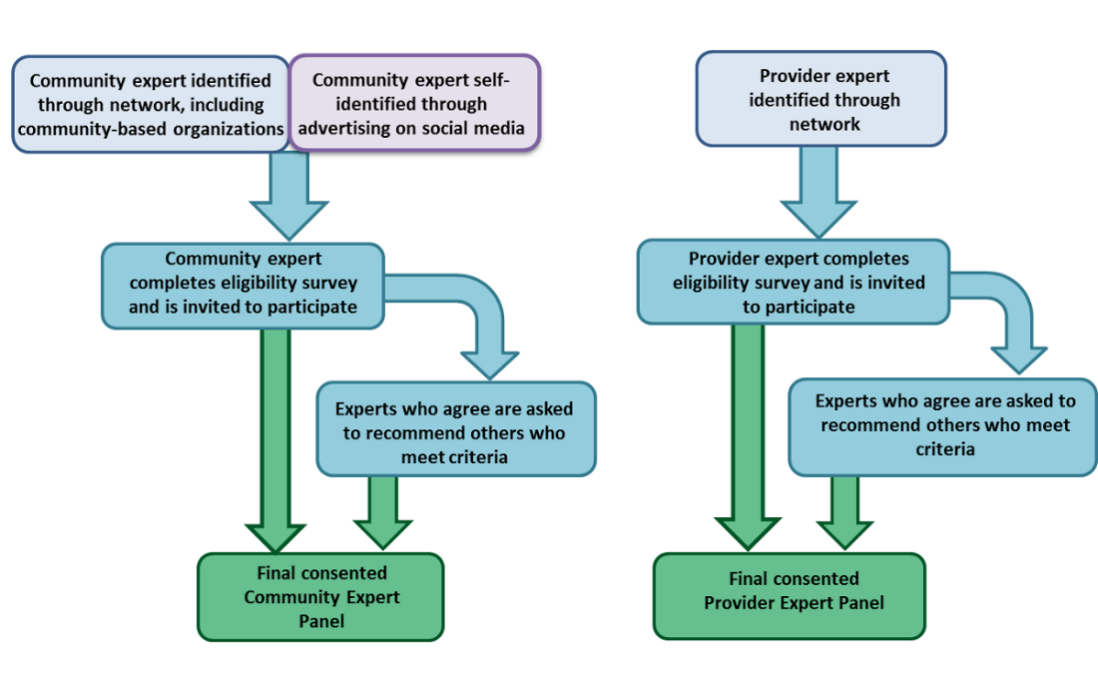
Flowchart for recruitment of community and provider expert panel. Left: To be eligible for the Community Panel, candidates (1) must be a cis- or trans-identified man aged 18 years and older, living in Toronto, and who has sex with men in the preceding 18 months and (2) must have sought and/or underwent sexually transmitted infection testing in Toronto in the preceding 18 months. Right: To be eligible for the Provider Panel, candidates must have a minimum of 1-year experience providing sexually transmitted infection testing and management care in Toronto.

The choice of experts for an initial invitation will be informed by our team’s community and professional networks. Experts who agree to participate will be encouraged to refer other eligible experts, mitigating potential bias from our team’s selection of members.

Using the approach shown in Figure 1, invitations will be informed by our team’s community and professional networks. Community participants will also be recruited via the existing social media channels (eg, Facebook and Twitter) of our community-based partner and through paid banner advertisements on popular gay dating apps (eg, Grindr). In addition, targeted emails will be sent to other organizations that serve the MSM community, including AIDS Service Organizations that cater to specific ethnoracial groups. These methods were successfully used in the recruitment of MSM for the focus groups and other studies conducted by our community-based partner [32]. Providers will be recruited using targeted emails to health care organizations known to serve large MSM patient populations, as we have done previously in our provider survey [33]. We aim to recruit a minimum of 30 experts with diverse backgrounds (including ethnoracial identity, gender identity, sexual orientation, and age) for each panel—feasible and sufficient for a Delphi study [2-4,35]. For community panelists, we have set target goals to recruit a minimum of 40% to be men aged ≤40 years and 40% to identify as non-white race/ethnicity.

Recruitment email invitations, social media, and dating app advertisements will include a link to an eligibility survey. Interested participants will need to complete the eligibility survey to identify those who meet the inclusion criteria. This step serves to minimize false participation. Eligible participants will then be sent a consent form to provide an email address or a phone number to receive the e-Delphi surveys. This information is not linked to the survey responses.

### E-Delphi Methods

The online surveys for each round of the e-Delphi will be delivered through Qualtrics (Provo, United States). Qualtrics is a secure Web-based survey platform and allows for anonymous participation. All data collected in Qualtrics will be stored in Canada and are protected with high-end firewalls and are treated confidentially. We will own and manage all the data collected via Qualtrics. All identified and interested members of the expert panel will be sent a personalized link to fill out each round of the survey. Although a personalized link will be used to access the survey, personal information will not be stored, and contact details will be removed in the completed survey.

#### Rounds

In the first round, panelists will review and consider the selected STI testing interventions. The preamble for each intervention will include a brief description and a list of considerations; panelists will also be given the opportunity to provide their opinion in an open text field. Panelists will be asked to rate each bacterial STI intervention on a 7-point Likert scale: 1=*definitely not a priority*, 2=*not a priority*, 3=*somewhat not a priority*, 4=*undecided*, 5=*somewhat of a priority*, 6=*a priority*, 7=*definitely a priority*. An open text field will be available for panelists to explain their priority choice. Finally, panelists will be invited to suggest an alternative STI testing intervention that was not listed but that they believe to be important. Panelists will also report their sociodemographic characteristics, specifically age, race/ethnicity, transgender identity, sexual orientation, and HIV serostatus (optional).

In the second round, panelists will be asked to prioritize the same interventions they considered in round 1. However, this time they will see the distribution of responses from the previous round (eg, the proportions of persons selecting each of the Likert scale options), as well as a summary of the rationale for prioritizing that particular intervention. Those who select a priority rating that does not agree with the majority will be asked to provide details for their choice with an open text field question, such as the following example: “Most guys chose an Online App for Booking Bacterial STI testing as ‘A priority’. Why did you not prioritize this option?”. Panelists will also be asked to rank their top 3 interventions that they consider the highest priority. If consensus is achieved after round 2, then that intervention option will be removed for round 3 prioritization (although they would still be included as options for respondents’ top 3 ranked interventions).

In round 3, panelists will again rate and rank the interventions alongside summaries of the prioritization and ranking responses from round 2, that is, they will have a third chance to rate interventions and a second chance to rank them. Those who rank a bacterial intervention component different from the majority will be asked to provide details for their choice with an open text field question: “One or more of your responses is a different priority than the other experts, please explain why you chose your response.”

#### Compensation

Each survey round will be accessible for 2 weeks, with 1-week breaks to conduct the analyses and provide response summaries for the subsequent round. To encourage retention throughout, we will provide increasing incentives at rates of Can $25, $35, and $40 for completion of rounds 1, 2, and 3 (total Can $100 for all 3 rounds), respectively. To receive this compensation, panelists will be provided with a link at the end of their survey which will take them to a reimbursement form to fill in contact information. The contact information will be collected and stored separately from study data and is asked for the purposes of reimbursement only.

#### Analysis

The analysis of responses from each round will occur iteratively and independently for the Community and Provider panels. The primary purpose is to achieve consensus within each of the panels to identify which subset of the proposed 8 interventions have the greatest potential for increasing testing levels among MSM in Toronto. As there is no standard definition of consensus for Delphi studies [35], we will define consensus as having ≥60% members (≥18/30) indicate a preference within 2 adjacent response points (+/−1) on a 7-point Likert scale. We will supplement the quantitative analyses with a thematic analysis of open-ended text data [36,37] to better understand disagreements within and between panels, should this occur. The top 3 ranked interventions will be determined based on frequency counts.

## Results

Progress to date includes knowledge synthesis and selection of candidate interventions for the e-Delphi surveys. In our updated literature review, we identified 246 publications, of which 88 were in the original published systematic reviews [24,34]. After applying our inclusion and exclusion criteria, 203 publications were excluded because of the following reasons: (1) the article did not describe an intervention aimed at increasing bacterial STI testing (n=176), (2) the intervention was implemented in a rural setting (n=1), (3) the study population included only women or heterosexual participants (n=22), and (4) study protocol of intervention (n=4).

In our final review, we included 43 publications describing 49 interventions. The largest number of publications were from Australia (n=15). Only 2 publications were from Canada. Effectiveness was categorized for these 49 interventions **(**Multimedia Appendix 1 **)**. A total of 37 interventions were deemed effective, with 24 moderately effective and 13 very effective.

In the category of streamlined testing among asymptomatic individuals, routine testing was the predominant intervention, with all 9 effective, followed by Web-based or home-based testing, with 6 out of 7 effective. A total of 8 effective interventions in this category incorporated testing of extragenital sites for chlamydia and gonorrhea, with 7 employing self-collection of anal swabs.

In the category of the client-targeted interventions, the most common intervention was client reminders, with 6 of 8 being effective, followed by 3 effective client counseling interventions. Both client incentive interventions (n=2) were ineffective.

In the category of the provider-targeted interventions, audit and feedback (n=2) and provider alerts (n=2) were effective. The effectiveness of provider education interventions was variable with 1 very effective and 1 ineffective study.

On the basis of the above evidence for effective interventions and emerging findings from our focus groups and provider survey, we selected the following interventions and their rationales for inclusion in the e-Delphi surveys (Table 2).

**Table 2:** Descriptions and rationale for interventions.

Category and intervention		Description	Rationale for inclusion	Summary of effectiveness
**Streamlined testing among asymptomatic individuals**				
	Routine testing	Clients are tested at every visit using standing orders.	Routine STI^a^ testing was effective in improving STI testing rates in all 9 studies identified by reducing stigma and normalizing testing.	Very effective: 5/10 studies [38-42]; Moderately effective: 4/10 studies [43-46]; Unknown effectiveness: 1/10 studies [47]
	Web-based/home-based testing	STI tests are ordered on the Web, client can opt for in-person lab testing or mailed self-testing kits.	Web-based or home testing was effective in improving STI testing rates in most studies, identified by increasing convenience and reducing the need to see a health care provider.	Very effective: 2/11 studies [48,49]; Moderately effective: 4/11 studies [50-53]; Ineffective: 1/11 studies [54]; Unknown: 4/11 studies [55-58]
	Nurse/nonphysician-led testing	A health care provider who is not a doctor (such as a nurse) collects information on a client’s sexual history and symptoms and collects samples.	A total of 2 identified studies demonstrated that having nurses provide testing is effective in improving STI testing rates with reducing the need to see a doctor and increased convenience.	Moderately effective: 2/2 studies [59]
	Express testing at clinics with self-collection of sample	On the basis of a self-completed questionnaire on sexual history and symptoms, clients are directed to self-collected testing if asymptomatic.	Express testing was effective in improving STI testing rates in 1 study by increasing convenience and reducing the need to see a health care provider.	Moderately effective: 1/2 studies (express clinic with self-collection of some specimens) [60], 1/2 studies (self-collection of samples in clinic) [61]
**Client-targeted**				
	Client reminders	Client gives permission to clinic to receive reminders via short message service text message, email, or mailed letter.	Client reminders were effective in improving STI testing rates in most studies identified. Clients are notified to test, and it becomes part of the health care routine.	Very effective: 4/9 studies [62-65]; Moderately effective: 3/9 studies [66-68]; Ineffective: 1/9 studies [69]; Unknown effectiveness: 1/9 studies [70]
	Web-based educational and testing booking app	Clients find information about bacterial STIs on an app/website and use it to book an appointment at a clinic.	A Web-based personally controlled health system manager was effective in improving STI testing rates by increasing knowledge and convenience.	Moderately effective: 1/1 study [71]
**Provider-targeted**				
	Provider audit and feedback	Providers receive a report on their own STI testing practices.	Providing feedback reports on STI testing rates was effective in improving STI testing rates by identifying good performance and areas to improve.	Very effective: 1/2 studies [72]; Moderately effective: 1/2 studies [73]
	Provider reminders	Providers receive alerts through electronic medical record systems to prompt an offer of STI testing.	Provider reminders to test clients at increased risk of STI acquisition were effective in improving STI testing rates by notifying provider to offer STI testing.	Moderately effective: 2/2 studies [74,75]

^a^STI: sexually transmitted infection.

## Discussion

### Overview

By conducting an e-Delphi exercise with community members and providers in Toronto, Canada, we will produce evidence to allow for community-directed, informed choices regarding the implementation of novel STI testing interventions for MSM. To maximize the chances for successful implementation, we first need to better understand the barriers to access testing and the intervention contexts in other settings, then work with community partners to determine which candidate intervention(s) would best overcome these barriers and how they may need to be adapted for the local context. Interventions must be acceptable to members of communities that they intend to serve [76]. Our choice of the e-Delphi method to prioritize potential interventions allows community members to have an equal voice alongside professional stakeholders.

Our plan is not without potential pitfalls. One challenge was selecting interventions for consideration by panelists. Our choices were based on an extensive literature review and qualitative and quantitative data on local patient and provider barriers and facilitators for STI testing. Nevertheless, it is possible that we overlooked or excluded interventions that could be effective in our setting. A second challenge is ensuring diversity in representation among members of the Community and Provider panels, as MSM communities are particularly heterogeneous in large urban cities, such as Toronto. We will seek out as representative a sample as possible to identify diverse perspectives but acknowledge that the opinions of panelists are unlikely to capture all possible views within a small sample size. Motivated panelists are crucial to ensure carefully considered ratings and high response and retention throughout the rounds. We will maximize input by limiting the number of questions asked and providing increasing incentives for completing each round. The potential for false participation is a concern (eg, participation by individuals pretending to meet the inclusion criteria), particularly for the establishment of the Community Panel. Procedures will minimize false participation including study promotion and direct invitations via established MSM community channels, an eligibility questionnaire step as we form the panel (without compensation), and a sliding scale of compensation, such that the highest amount is provided for completion of the third and final questionnaire. Finally, consensus may not be reached at the end of the 3 rounds within and between each expert panel. However, in conducting the Delphi panels, we will gain a better understanding of the interventions with the greatest potential for improving local STI testing services for MSM in Toronto and be better positioned to anticipate potential roadblocks to implementation.

### Conclusions

Innovative approaches to health care delivery are needed to produce the required increases in bacterial STI testing levels, frequency, and the use of appropriate testing technologies in ways that are engaging, nonstigmatizing, and acceptable for MSM [21]. Many community- and clinic-based bacterial STI test interventions have demonstrated effectiveness in the international literature [24,34] and/or are being attempted as pilot projects in Canada [22,55]. Yet the choice of intervention to implement can be daunting without local evidence regarding the best fit. The results of the proposed e-Delphi will characterize the intervention climate including perceived needs for changes to test delivery, relative priorities for change, and readiness for implementation [77]. Our approach may be transferable to other settings where stakeholder input is needed to manage sensitive areas of concern.

## Appendix

Multimedia Appendix 1 Effectiveness of 49 interventions from 43 articles.

Multimedia Appendix 2 Peer-reviewer report from the Canadian Institutes of Health Research.

## Abbreviations

AD: absolute difference
CIHR: Canadian Institutes of Health Research
MSM: men who have sex with men
RD: relative difference
STI: sexually transmitted infection
