# Supplementary material for: Community-Directed Bacterial Sexually Transmitted Infection Testing Interventions Among Men Who Have Sex With Men: Protocol for an E-Delphi Study in Toronto, Canada
Source: JMIR Res Protoc. 2019 Jul 4;8(7):e13801. doi: 10.2196/13801 (PMC6637728; doi:10.2196/13801)
Supplement: Multimedia Appendix 1 [file resprot_v8i7e13801_app1.pdf]

Supplement 1: Effectiveness of 49 interventions from 43 publications.

The updated literature review identified 43 publications describing 49 interventions. These were categorized into 3 groups: 1) Streamlined testing among asymptomatic individuals 2) Client-targeted interventions and 3) provider-targeted interventions. Each intervention was then classified by effectiveness (very effective, moderately effective, ineffective and unknown.. The size of each intervention circle reflects the sample size of the study.

Unless otherwise indicated, all studies used Absolute Difference (AD) to evaluate effectiveness. The AD was calculated as change in percent screened (percent screened in intervention group minus percent screened in comparison group)

(a) The Relative Difference was used to evaluate effectiveness, calculated as the relative percent change in tests performed [(number of tests or patients tested in intervention group minus tests or patients tested in comparison group)/number of tests or patients tested in the comparison group)*100].

All studies included male participants. Those that specifically included gay, bisexual and other men who have sex with men (MSM), MSM living with HIV, and/or MSM who were HIV-negative are indicated as shown.

(b) The sample population included some or only MSM.

(+) Sample population included some or only men living with HIV.

(-) Sample population only included those not living with HIV.

Studies marked in red are not included in e-Delphi studies. Intervention types not included in e-Delphi studies are Targeted Screening, Client Counselling, Client Education, Outreach and Provider Education.

Studies numbered XX.1 (ie 27.1) indicate that different populations were evaluated for intervention.

Figure 1: Effectiveness of Interventions

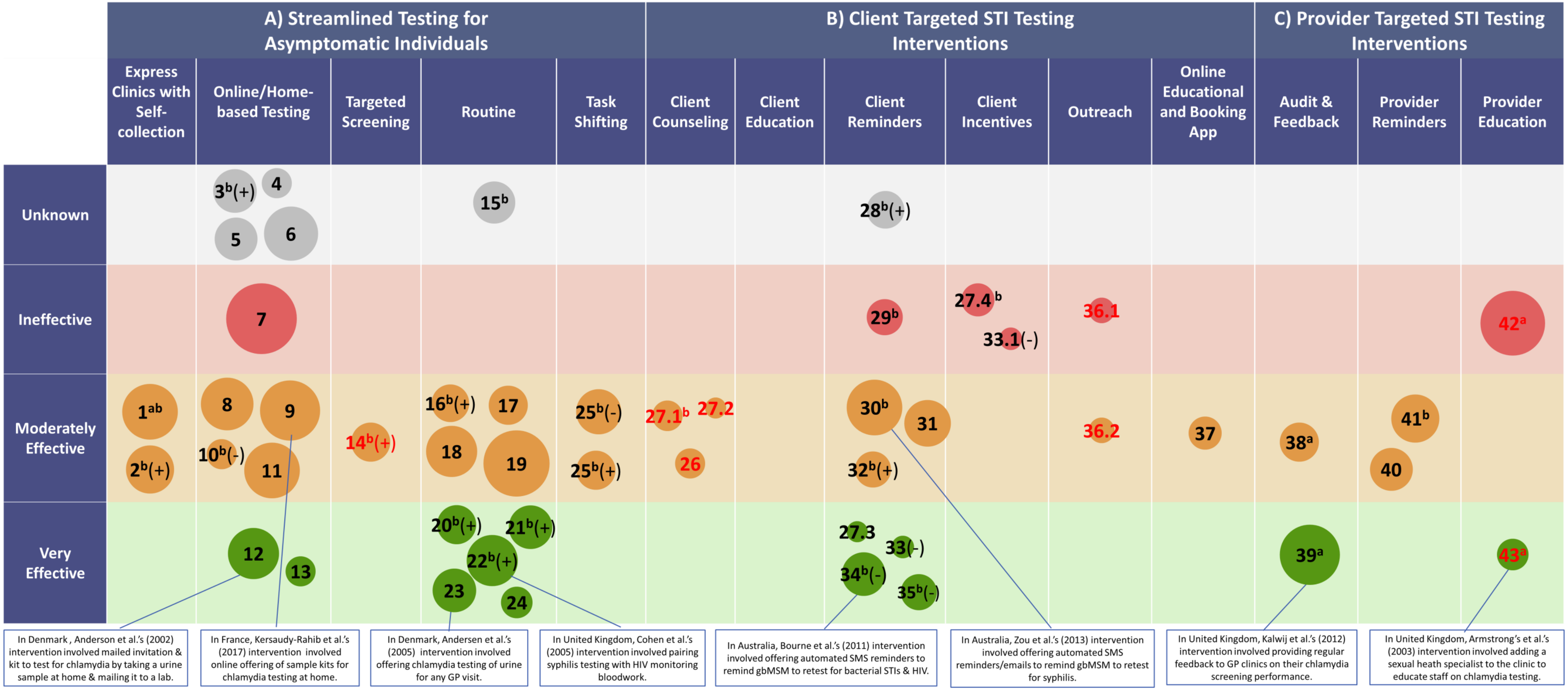

References

1. Barbee LA, Tat S, Dhanireddy S,Marrazzo JM. *Implementation and Operational Research: Effectiveness and Patient Acceptability of a Sexually Transmitted Infection Self-Testing Program in an HIV Care Setting.* J Acquir Immune Defic Syndr, 2016. **72**(2): p. e26-e31. DOI: 10.1097/QAI.0000000000000979 PMID:26959189
2. Knight V, Ryder N, Guy R, Lu H, Wand H,McNulty A. *New Xpress Sexually Transmissible Infection Screening Clinic Improves Patient Journey and Clinic Capacity at a Large Sexual Health Clinic.* Sex Transm Dis, 2013. **40**(1): p. 75-80. DOI: 10.1097/OLQ.0b013e3182793700 PMID:23250305
3. Gilbert M, Salway T, Haag D, Fairley CK, Wong J, Grennan T, Uddin Z, Buchner CS, Wong T, Krajden M, Tyndall M, S. J,Ogilvie G. *Use of GetCheckedOnline, a Comprehensive Web-based Testing Service for Sexually Transmitted and Blood-Borne Infections.* J Med Internet Res, 2017. **19**(3): p. e81-e81. DOI: 10.2196/jmir.7097 PMID:28320690
4. Mann TA, Uddin Z, Hendriks AM, Bouchard CJ,Etches VG. *Get Tested Why Not? A Novel Approach to Internet-based Chlamydia and Gonorrhea Testing in Canada.* Can J Public Health, 2013. **104**(3): p. e205-e209. DOI: 10.17269/cjph.104.3684 PMID:23823883
5. Kwan KSH, Jachimowicz EA, Bastian L, Marshall L,Mak DB. *Online chlamydia testing: an innovative approach that appeals to young people.* Med J Aust, 2012. **197**(5): p. 287-290. DOI: https://www.ncbi.nlm.nih.gov/pubmed/22938127 PMID:22938127
6. Götz HM, van den Broek IVF, Hoebe CJPA, Brouwers EEHG, Pars LL, Fennema JSA, Koekenbier RH, van Ravesteijn SM, Op de Coul ELM,van Bergen JEAM. *High yield of reinfections by home-based automatic rescreening of Chlamydia positives in a large-scale register-based screening programme and determinants of repeat infections.* Sex Transm Infect, 2013. **89**(1): p. 63-69. DOI: 10.1136/sextrans-2011-050455 PMID:22728910
7. van den Broek IVF, van Bergen JEAM, Brouwers EEHG, Fennema JSA, Götz HM, Hoebe CJPA, Koekenbier RH, Kretzschmar M, Over EAB, Schmid BV, Pars LL, van Ravesteijn SM, van der Sande MAB, de Wit GA, Low N,Op de Coul ELM. *Effectiveness of yearly, register based screening for chlamydia in the Netherlands: controlled trial with randomised stepped wedge implementation.* BMJ, 2012. **345**: p. e4316. DOI: 10.1136/bmj.e4316 PMID:22767614
8. Scholes D, Heidrich FE, Yarbro P, Lindenbaum JE,Marrazzo JM. *Population-based outreach for Chlamydia screening in men: results from a randomized trial.* Sex Transm Dis, 2007. **34**(11): p. 837-839. DOI: 10.1097/OLQ.0b013e31805ba860 PMID:17538514
9. Kløvstad H, Natås O, Tverdal A,Aavitsland P. *Systematic screening with information and home sampling for genital Chlamydia trachomatis infections in young men and women in Norway: a randomized controlled trial.* BMC Infect Dis, 2013. **13**(1): p. 30. DOI: 10.1186/1471-2334-13-30 PMID:23343391
10. Smith KS, Hocking JS, Chen MY, Fairley CK, McNulty AM, Read P, Bradshaw CS, Tabrizi SN, Wand H, Saville M, Rawlinson W, Garland SM, Donovan B, Kaldor JM,Guy RJ. *Dual Intervention to Increase Chlamydia Retesting. A Randomized Controlled Trial in Three Populations.* Am J Prev Med, 2015. **49**(1): p. 1-11. DOI: 10.1016/j.amepre.2015.01.014 PMID:S0749379715000343
11. Kersaudy-Rahib D, Lydié N, Leroy C, March L, Bébéar C, A. P,de Barbeyrac B. *Chlamyweb Study II: a randomised controlled trial (RCT) of an online offer of home-based Chlamydia trachomatis sampling in France.* Sex Transm Infect, 2017. **93**(3): p. 188-195. DOI: 10.1136/sextrans-2015-052510 PMID:28377422
12. Andersen B, Olesen F, Møller JK,Østergaard L. *Population-Based Strategies for Outreach Screening of Urogenital Chlamydia trachomatis Infections: A Randomized, Controlled Trial.* J Infect Dis, 2002. **185**(2): p. 252-258. DOI: 10.1086/338268 PMID:11807700
13. Reagan MM, Xu H, Shih SL, Secura GM,Peipert JF. *A Randomized Trial of Home Versus Clinic-Based Sexually Transmitted Disease Screening Among Men.* Sex Transm Dis, 2012. **39**(11): p. 842-847. DOI: 10.1097/OLQ.0b013e3182649165 PMID:23064532
14. Scarborough AP, Slome S, Hurley LB,Park IU. *Improvement of sexually transmitted disease screening among HIV-infected men who have sex with men through implementation of a standardized sexual risk assessment tool.* Sex Transm Dis, 2015. **42**(10): p. 595. PMID:26372932
15. Ritchie S, Henley R, Hilton J, Handy R, Ingram J, Mundt S, Nisbet M, Thomas M,Briggs S. *Uptake, yield and resource requirements of screening for asymptomatic sexually transmissible infections among HIV-positive people attending a hospital outpatient clinic.* Sex Health, 2014. **11**(1): p. 67-72. DOI: https://doi.org/10.1071/SH13167 PMID:24618022
16. Botes LP, McAllister J, Ribbons E, Jin F,Hillman RJ. *Significant increase in testing rates for sexually transmissible infections following the introduction of an anal cytological screening program, targeting HIV-positive men who have sex with men.* Sex Health, 2011. **8**(1): p. 76-8. DOI: 10.1071/sh10027 PMID:21371387

17. Lawton BA, Rose SB, Elley CR, Bromhead C, MacDonald EJ,Baker MG. *Increasing the uptake of opportunistic chlamydia screening: a pilot study in general practice.* J Prim Health Care, 2010. **2**(3): p. 199-207. DOI: https://www.ncbi.nlm.nih.gov/pubmed/21069115 PMID:21069115
18. Graham S, Guy RJ, Wand HC, Kaldor JM, Donovan B, Knox J, McCowen D, Bullen P, Booker J, O'Brien C, Garrett K,Ward JS. *A sexual health quality improvement program (SHIMMER) triples chlamydia and gonorrhoea testing rates among young people attending Aboriginal primary health care services in Australia.* BMC Infect Dis, 2015. **15**(1): p. 370. DOI: 10.1186/s12879-015-1107-5 PMID:26329123
19. Patton ME, Kirkcaldy RD, Chang DC, Markman S, Yellowman M, Petrosky E, Adams L, Robinson C, Gupta A,Taylor MM. *Increased gonorrhea screening and case finding after implementation of expanded screening criteria--Urban Indian Health Service Facility in Phoenix, Arizona, 2011-2013.* Sex Transm Dis, 2016(6): p. 396. DOI: 10.1097/OLQ.0000000000000457 PMID:27200523
20. Bissessor M, Fairley CK, Leslie D, Howley K,Chen MY. *Frequent screening for syphilis as part of HIV monitoring increases the detection of early asymptomatic syphilis among HIV-positive homosexual men.* J Acquir Immune Defic Syndr, 2010. **55**(2): p. 211-216. DOI: 10.1097/QAI.0b013e3181e583bf PMID:20585261
21. Callander D, Baker D, Chen M,Guy R. *Including Syphilis Testing as Part of Standard HIV Management Checks and Improved Syphilis Screening in Primary Care.* Sex Transm Dis, 2013(4): p. 338-340. DOI: 10.1097/OLQ.0b013e31828052c5 PMID:23486501
22. Cohen CE, Winston A, Asboe D, Boag F, Mandalia S,Azadian B. *Increasing detection of asymptomatic syphilis in HIV patients.* Sex Transm Infect, 2005. **81**(3): p. 217-219. DOI: 10.1136/sti.2004.012187 PMID:15923288
23. Andersen B, Eidner PO, Hagensen D, Lomborg S,Hoff G. *Opportunistic screening of young men for urogenital Chlamydia trachomatis infection in general practice.* Scand J Infect Dis, 2005. **37**(1): p. 35-39. DOI: 10.1080/00365540510026418 PMID:15764188
24. Tebb KP, Pantell RH, Wibbelsman CJ, Neuhaus JM, Tipton AC, Pecson SC, Pai-Dhungat M, Ko TH,Shafer MB. *Screening sexually active adolescents for Chlamydia trachomatis: what about the boys?* Am J Public Health, 2005. **95**(10): p. 1806-1810. DOI: 10.2105/AJPH.2003.037507 PMID:16186459
25. Snow AF, Vodstrcil LA, Fairley CK, El-Hayek C, Cummings R, Owen L, Roth N, Hellard ME,Chen MY. *Introduction of a sexual health practice nurse is associated with increased STI testing of men who have sex with men in primary care.* BMC Infect Dis, 2013. **13**(1): p. 298. DOI: 10.1186/1471-2334-13-298 PMID:89638677
26. Kang M, Rochford A, Skinner R, Mindel A, Webb M, Peat J,Usherwood T. *Facilitating chlamydia testing among young people: a randomised controlled trial in cyberspace.* Sex Transm Infect, 2012. **88**(8): p. 568-573. DOI: 10.1136/sextrans-2011-050124 PMID:22764218
27. Malotte CK, Ledsky R, Hogben M, Larro M, Middlestadt S, StLawrence JS, Olthoff G, Settlage RH, Van Devanter NL,Gcap Study Group. *Comparison of Methods to Increase Repeat Testing in Persons Treated for Gonorrhea and/or Chlamydia at Public Sexually Transmitted Disease Clinics.* Sex Transm Dis, 2004. **31**(11): p. 637-42. https://www.ncbi.nlm.nih.gov/pubmed/15502669 PMID:15502669
28. Harte D, Mercey D, Jarman J,Benn P. *Is the recall of men who have sex with men (MSM) diagnosed as having bacterial sexually transmitted infections (STIs) for re-screening a feasible and effective strategy?* Sex Transm Infect, 2011. **87**(7): p. 577-582. DOI: 10.1136/sextrans-2011-050144 PMID:21965470
29. Burton J, Brook G, McSorley J,Murphy S. *The utility of short message service (SMS) texts to remind patients at higher risk of STIs and HIV to reattend for testing: a controlled before and after study.* Sex Transm Infect, 2014. **90**(1): p. 11-13. DOI: 10.1136/sextrans-2013-051228 PMID:24064987
30. Zou H, Fairley CK, Guy R, Bilardi J, Bradshaw CS, Garland SM, Sze JK, Afrizal A,Chen MY. *Automated, Computer Generated Reminders and Increased Detection of Gonorrhoea, Chlamydia and Syphilis in Men Who Have Sex with Men.* PLOS ONE, 2013. **8**(4): p. e61972. DOI: 10.1371/journal.pone.0061972 PMID:23613989
31. Paneth-Pollak R, Klingler EJ, Blank S,Schillinger JA. *The Elephant Never Forgets; Piloting a Chlamydia and Gonorrhea Retesting Reminder Postcard in an STD Clinic Setting.* Sex Transm Dis, 2010. **37**(6): p. 365-8. DOI: 10.1097/OLQ.0b013e3181cab281 PMID:20473247
32. Fernando KA, Fowler T, Harding J, Flew S, Caley M, Phattey J,Ross JDC. *Detecting re-infection in patients after an initial diagnosis of gonorrhoea: is routine recall for re-screening useful?* Int J STD AIDS, 2015. **26**(9): p. 640-647. DOI: 10.1177/0956462414548905 PMID:25161175

33. Downing SG, Cashman C, McNamee H, Penney D, Russell DB,Hellard ME. *Increasing chlamydia test of re-infection rates using SMS reminders and incentives.* Sex Transm Infect, 2013. **89**(1): p. 16-19. DOI: 10.1136/sextrans-2011-050454 PMID:22728911
34. Bourne C, Knight V, Guy R, Wand H, Lu H,McNulty A. *Short message service reminder intervention doubles sexually transmitted infection/HIV re-testing rates among men who have sex with men.* Sex Transm Infect, 2011. **87**(3): p. 229-31. DOI: 10.1136/sti.2010.048397 PMID:21296796
35. Nyatsanza F, McSorley J, Murphy S,Brook G. *'It's all in the message': the utility of personalised short message service (SMS) texts to remind patients at higher risk of STIs and HIV to reattend for testing—a repeat before and after study.* Sex Transm Infect, 2016. **92**(5): p. 393-395. DOI: 10.1136/sextrans-2015-052216 PMID:26670912
36. Jackson LJ, Roberts TE, Fuller SS, Sutcliffe LJ, Saunders JM, Copas AJ, Mercer CH, Cassell JA,Estcourt CS. *Exploring the costs and outcomes of sexually transmitted infection (STI) screening interventions targeting men in football club settings: preliminary cost-consequence analysis of the SPORTSMART pilot randomised controlled trial.* Sex Transm Infect, 2015. **91**(2): p. 100-105. DOI: 10.1136/sextrans-2014-051715 PMID:25512670
37. Mortimer NJ, Rhee J, Guy R, Hayen A,Lau AYS. *A web-based personally controlled health management system increases sexually transmitted infection screening rates in young people: a randomized controlled trial.* JAMIA, 2015. **22**(4): p. 805-814. DOI: 10.1093/jamia/ocu052 PMID:25773130
38. Merritt TD, Durrheim DN, Hope K,Byron P. *General practice intervention to increase opportunistic screening for chlamydia.* Sex Health, 2007. **4**(4): p. 249-251. DOI: doi:10.1071/SH07033 PMID:18082068
39. Kalwij S, French S, Mugezi R,Baraitser P. *Using educational outreach and a financial incentive to increase general practices' contribution to chlamydia screening in South-East London 2003-2011.* BMC Public Health, 2012. **12**: p. 802. DOI: 10.1186/1471-2458-12-802 PMID:22984897
40. Lister NA, Smith A,Fairley CK. *Introduction of screening guidelines for men who have sex with men at an STD clinic, the Melbourne Sexual Health Centre, Australia.* Sex Health, 2005. **2**(4): p. 241-244. https://www.ncbi.nlm.nih.gov/pubmed/16402672 PMID:16402672
41. Bissessor M, Fairley CK, Leslie D,Chen MY. *Use of a Computer Alert Increases Detection of Early, Asymptomatic Syphilis Among Higher-Risk Men Who Have Sex With Men.* Clin Infect Dis, 2011. **53**(1): p. 57-58. DOI: 10.1093/cid/cir271 PMID:21653303
42. Town K, McNulty CA, Ricketts EJ, Hartney T, Nardone A, Folkard KA, Charlett A,Dunbar JK. *Service evaluation of an educational intervention to improve sexual health services in primary care implemented using a step-wedge design: analysis of chlamydia testing and diagnosis rate changes.* BMC Public Health, 2016. **16**: p. 686. DOI: 10.1186/s12889-016-3343-z PMID:27484823
43. Armstrong B, Kinn S, Scoular A,Wilson P. *Shared care in the management of genital Chlamydia trachomatis infection in primary care.* Sex Transm Infect, 2003. **79**(5): p. 369-371. DOI: https://www.ncbi.nlm.nih.gov/pmc/articles/PMC1744748/ PMID:14573830
