# Supplementary material for: Community-Directed Bacterial Sexually Transmitted Infection Testing Interventions Among Men Who Have Sex With Men: Protocol for an E-Delphi Study in Toronto, Canada
Source: JMIR Res Protoc. 2019 Jul 4;8(7):e13801. doi: 10.2196/13801 (PMC6637728; doi:10.2196/13801)
Supplement: Multimedia Appendix 2 [file resprot_v8i7e13801_app2.pdf]

October/Octobre 20, 2016

DC0190GP

Canadian Institutes of Health Research / Instituts de recherche en santé du Canada

Notice of Decision / Avis de décision

Application Number/Numéro de la demande: 372763

Committee Code/Code du comité: CBF

Applicants/Candidats: Dr. Ann Natalie Burchell   Mr. Ryan Charles Lisk

With/Avec:				
	Ms. J. Bacon	Dr. J. Brunetta	Dr. D. Gesink	Dr. M. Gilbert
	Ms. R. Grewal	Dr. C. Guiang	Mr. M. Kwag	Dr. C. Logie
	Mr. L. Mitterni	Dr. R. Shahin	Dr. D. Tan	

Institution paid/ Établissement payé: St. Michael's Hospital (Toronto)

Title/Titre: Bringing bacterial STIs to the agenda for comprehensive sexual health care: Community-directed interventions to facilitate testing among people living with and at risk for HIV

Primary Inst./ Inst. principal: Health Services and Policy Research

Other Related Inst./ Autres inst. connexes: Population and Public Health; Gender and Health

Competition Outcome/Résultats du concours: Catalyst Grant: HIV/AIDS CBR Program - General Stream
June/Juin 28, 2016

Number in competition/Nbre de demandes dans le concours: 21

Number approved/Nbre de demandes approuvées: 6

Decision on your application/ Décision sur votre demande: Approved

Average annual amount/ Montant annuel moyen: $32,970

Equipment amount/ Montant pour les appareils: $0

Term/Durée: 1 **yrs/ans** 0 **months/mois**

Peer Review Committee Recommendation, for your information and use/ Recommandation du comité d'examen par les pairs, pour fins d'information et d'utilisation:

Committee/Comité: HIV/AIDS Community-Based Research (merged)

Application rank within the competition/ Rang de la demande dans ce concours: 4

Percent Rank Within the Competition/ Rang en pourcentage au sein du concours: 19.05%

Rating/ Cote: 4.195

Recommended average annual amount/ Montant annuel moyen recommandé: $32,970

Recommended equipment amount/ Montant recommandé pour les appareils: $0

*** Applications receiving a score of less than 3.5 on any evaluation criteria will not be considered for Funding. / Les demandes qui ont reçu une note inférieure à 3.5 pour n'importe quel des critères d'évaluation ne sont pas admissibles.

This document is for information only. Official payment is stated on the CIHR Authorization for Funding.
Document à titre d'information seulement. Le paiement officiel est indiqué sur l'autorisation de financement des IRSC.

CIHR IRSC

Institute of Aboriginal Peoples' Health
Institute of Aging
Institute of Cancer Research
Institute of Circulatory and Respiratory Health
Institute of Gender and Health
Institute of Genetics
Institute of Health Services and Policy Research
Institute of Human Development and Child and Youth Health
Institute of Infection and Immunity
Institute of Musculoskeletal Health and Arthritis
Institute of Neurosciences, Mental Health and Addiction
Institute of Nutrition, Metabolism and Diabetes
Institute of Population and Public Health

Institut de la santé des Autochtones
Institut du vieillissement
Institut du cancer
Institut de la santé circulatoire et respiratoire
Institut de la santé des femmes et des hommes
Institut de génétique
Institut des services et des politiques de la santé
Institut du développement et de la santé des enfants et des adolescents
Institut des maladies infectieuses et immunitaires
Institut de l'appareil locomoteur et de l'arthrite
Institut des neurosciences, de la santé mentale et des toxicomanies
Institut de la nutrition, du métabolisme et du diabète
Institut de la santé publique et des populations

October 20, 2016

Dr. Ann Natalie Burchell
St. Michael's Hospital
Department of Family and Community Medicine
Li Ka Shing Knowledge Institute
30 Bond Street
Toronto, Ontario M5B 1W8

Dear Dr. Burchell,

On behalf of the Canadian Institutes of Health Research (CIHR) HIV/AIDS Research Initiative and its partners, we are pleased to inform you that your recent grant application entitled “Bringing bacterial STIs to the agenda for comprehensive sexual health care: Community-directed interventions to facilitate testing among people living with and at risk for HIV”, submitted to the Catalyst Grant: HIV/AIDS CBR Program - General Stream funding opportunity, has been approved for funding. The HIV/AIDS Research Initiative and its programs are components of the Federal Initiative to Address HIV/AIDS in Canada.

You will find the review documents related to your proposal in your ResearchNet account. If you cannot access the review documents related to your proposal through ResearchNet, please contact CIHR. The Authorization for Funding will be mailed to you shortly. The Scientific Officer Notes and reviewers' reports are provided in the language in which they were written. If you would like them to be translated, please contact us and we will be pleased to do so.

As CIHR does not notify co-applicants by mail of the decision taken, we ask that you inform the other individuals involved in this project along with their research institutions (if different from your own) of the outcome of this application.

Should you have any questions about the review process, please address them directly to support@cihr-irsc.gc.ca. Please do not contact the officers or members of the merit review committee.

Congratulations on your success in this competition!

Sincerely,

L. McKenzie

Linda McKenzie
Manager, Program Design and Delivery
Research, Knowledge Translation Portfolio and Ethics Portfolio

440612-201606CDE-CBF-372763-35546-LKJHL

CIHR IRSC

Institute of Aboriginal Peoples' Health
Institute of Aging
Institute of Cancer Research
Institute of Circulatory and Respiratory Health
Institute of Gender and Health
Institute of Genetics
Institute of Health Services and Policy Research
Institute of Human Development and Child and Youth Health
Institute of Infection and Immunity
Institute of Musculoskeletal Health and Arthritis
Institute of Neurosciences, Mental Health and Addiction
Institute of Nutrition, Metabolism and Diabetes
Institute of Population and Public Health

Institut de la santé des Autochtones
Institut du vieillissement
Institut du cancer
Institut de la santé circulatoire et respiratoire
Institut de la santé des femmes et des hommes
Institut de génétique
Institut des services et des politiques de la santé
Institut du développement et de la santé des enfants et des adolescents
Institut des maladies infectieuses et immunitaires
Institut de l'appareil locomoteur et de l'arthrite
Institut des neurosciences, de la santé mentale et des toxicomanies
Institut de la nutrition, du métabolisme et du diabète
Institut de la santé publique et des populations

October 20, 2016

Dr. Ann Natalie Burchell
St. Michael's Hospital
Department of Family and Community Medicine
Li Ka Shing Knowledge Institute
30 Bond Street
Toronto, Ontario M5B 1W8

Dear Dr. Burchell:

Congratulations on your success in the recent Canadian Institutes of Health Research (CIHR) funding competition.

Canadian health researchers are recognized worldwide for their inspiration, curiosity, and drive. As a CIHR-funded researcher, you are helping to build this reputation and carry on Canada’s tradition of scientific excellence. As you pursue your research project, it is our hope that your efforts will help create new knowledge that will improve health and strengthen the health care system for the benefit of Canadians.

As you know, peer review is the cornerstone of our research funding system. This process is made possible because of the kind volunteerism of individuals who generously gave their time to review your application. As a CIHR-funded researcher, you may be invited to serve in the peer review process as we are continuously recruiting and retaining the most accomplished, innovative and creative scientists to review health research proposals.

To meet CIHR goals, we must also continue to communicate the value of health research to Canadians. That is why we encourage you to work with CIHR to promote your research. We have developed guidelines on public communication, available at: www.cihr-irsc.gc.ca/e/30789.html, to support you in this activity.

Once again, I offer you my congratulations and best wishes for success in your research.

Yours sincerely,

Alain Beaudet, MD, Ph.D.
President

440613-201606CDE-CBF-372763-35546-CONGR

President

Canadian Institutes of Health Research
Room 97, 160 Elgin Street, Address locator: 4809A
Ottawa, (Ontario) K1A 0W9 Tel.: (613) 941-2672
Fax (613) 954-1800 **www.cihr-irsc.gc.ca**

Président

Instituts de recherche en santé du Canada
Pièce 97, 160 rue Elgin, Indice de l'adresse: 4809A
Ottawa, (Ontario) K1A 0W9 Tél.: (613) 941-2672
Fax (613) 954-1800 **www.irsc-cihr.gc.ca**

Canada

Application Number / Numéro de demande: 371407
Name of Applicant / Nom du chercheur: Burchell, Ann Natalie
Review Type / Type d'évaluation: Committee Member 1/Membre de comité 1
Competition: 2016-06-28 Catalyst Grant: HIV/AIDS Community Based Research
Concours: 2016-06-28 Subvention catalyseur : recherche communautaire sur le VIH/sida
Committee: HIV/AIDS Community-Based Research (merged)
Comité: Recherche communautaire sur le VIH/SIDA (fusionné)

Potential Impact

Comments:

This project is important and timely given the advances in HIV treatment that lead to undetectable viral loads, which likely leads to less condom use. More attention to bacterial STIs is warranted. Efforts to increase testing are needed. Knowledge on which interventions should be implemented among MSM in Toronto will be valuable. The urgent need for this type of intervention was identified by the STI prevention community.

Although the initiative is indirectly linked to HIV, it addresses an issue that is relevant to HIV work. Applicants situate this well in their 'Contribution' section: "Further, our work will lead directly to the choice of STI testing interventions for implementation, thereby addressing the HIV care continuum in line with the CIHR HIV/AIDS Research Initiative’s Strategic Plan."

Regarding their CBR Principles, the team does not clearly indicate that it includes members of Toronto's MSM community.

**Application Number / Numéro de demande:**	371407
**Name of Applicant / Nom du chercheur:**	Burchell, Ann Natalie
**Review Type / Type d'évaluation:**	Committee Member 1/Membre de comité 1
**Competition:**	2016-06-28 Catalyst Grant: HIV/AIDS Community Based Research
**Concours:**	2016-06-28 Subvention catalyseur : recherche communautaire sur le VIH/sida
**Committee:**	HIV/AIDS Community-Based Research (merged)
**Comité:**	Recherche communautaire sur le VIH/SIDA (fusionné)

Scientific Merit

Comments:

The goal and objectives of this proposed project are clear and simple, with a manageable scope for a catalyst grant.

The proposal is very well written and clearly situates the problem and research question.

The methods are also clear and well described and appropriate for what they want to achieve. However, the applicants describe that they will be conducting an online modified Delphi study. The project could probably benefit from a face-to-face discussion of the Expert Panel for the Third Delphi round to achieve consensus on the interventions. The CBR Principles Summary only describes that the team will share the project findings with the Expert Panel members and other community stakeholders.

I wonder about the diversity of the MSM population in Toronto and whether this diversity has been documented. The application could benefit from describing how this diversity will be represented on the expert panel. Various needs in the community might affect key characteristics of potential interventions. A bit more information on this would be useful.

The team has a good KT plan.

**Application Number / Numéro de demande:**	371407
**Name of Applicant / Nom du chercheur:**	Burchell, Ann Natalie
**Review Type / Type d'évaluation:**	Committee Member 2/Membre de comité 2
**Competition:**	2016-06-28 Catalyst Grant: HIV/AIDS Community Based Research
**Concours:**	2016-06-28 Subvention catalyseur : recherche communautaire sur le VIH/sida
**Committee:**	HIV/AIDS Community-Based Research (merged)
**Comité:**	Recherche communautaire sur le VIH/SIDA (fusionné)

Potential Impact

Comments:

Summary
In Canada, HIV-positive and –negative men who have sex with men (MSM) have experienced dramatic rises in bacterial sexually transmitted infections (STIs)—syphilis, gonorrhea, and chlamydia. STI testing and treatment mitigate adverse health outcomes and substantially reduce transmission, yet testing rates remain below recommended levels. One-off "testing blitzes" have not led to frequent testing, resulting in rebounds and rising infection rates. Few undergo recommended extragenital testing for gonorrhea and chlamydia. Without comprehensive sexual health promotion, advances in HIV medicine may unintentionally fuel STI transmission. Innovation is needed to produce the required increases in test coverage, frequency, and use of appropriate testing technologies in ways that are engaging, non-stigmatizing, and acceptable.

Objectives and Approach
To answer our question, "Which bacterial STI testing interventions should be implemented and evaluated among MSM communities in Toronto?" we will:
1-Synthesize knowledge regarding current test programs and practice; barriers and facilitators of testing among MSM; and evidence regarding effectiveness for novel test interventions; and
2-Form an Expert Panel of community members, knowledge users, and researchers for a modified Delphi study to build consensus regarding intervention(s) with the greatest potential for our setting.

Outcomes
We will identify STI testing interventions most adaptable to our setting according to MSM community members, healthcare providers, and health systems experts, laying the groundwork for rigorous evaluation via a future grant proposal. Our project will lead to community-directed, informed decision making regarding STI test delivery in urban settings that may guide other jurisdictions experiencing similar epidemics.

Comments
Potential Impact #1: The application responds to the objectives and relevant research areas of the funding opportunity.
Potential Impact #2: The research team has already developed strong collaborations through previous projects.
Potential Impact #3: The activities of iKTE are very structured and, if their analysis is adequate (see Scientific Merit), they will have a major impact on the communities concerned by STI and HIV.

The proposal specifically addresses the objectives of this funding opportunity because it is catalytic in nature, bringing together community members, front-line providers, clinicians and researchers to generate and synthesize evidence for STI testing interventions for MSM within a CBR framework. The work will lead directly to the choice of STI testing interventions for implementation, thereby addressing the HIV care continuum in line with the CIHR HIV/AIDS Research Initiative's Strategic Plan.

Application Number / Numéro de demande: 371407
Name of Applicant / Nom du chercheur: Burchell, Ann Natalie
Review Type / Type d'évaluation: Committee Member 2/Membre de comité 2
Competition: 2016-06-28 Catalyst Grant: HIV/AIDS Community Based Research
Concours: 2016-06-28 Subvention catalyseur : recherche communautaire sur le VIH/sida
Committee: HIV/AIDS Community-Based Research (merged)
Comité: Recherche communautaire sur le VIH/SIDA (fusionné)

Scientific Merit

Comments:

The problematic is extremely important and the impact of the project is obvious. There is no doubt on the experience of the research team which is extremely solid.

Background and rationale are very well documented. The combined prevention is very well described along with the gaps to fulfill.

Methods are realistic and timeline feasible.

This project would allow further grant proposal in order to solve the problematic of STI and MSM.

Budget and Budget Justifications: No comment, perfectly realistic.

This project should be financed as it is.
